# Revealing the crosstalk between nasopharyngeal carcinoma and immune cells in the tumor microenvironment

**DOI:** 10.1186/s13046-022-02457-4

**Published:** 2022-08-13

**Authors:** Jianyun Jiang, Hongmei Ying

**Affiliations:** 1 Department of Radiation Oncology, Fudan University Shanghai Cancer Center, Shanghai, 200032 China; 2 Department of Oncology, Shanghai Medical College, Fudan University, Shanghai, 200032 China; 3 Shanghai Key Laboratory of Radiation Oncology, Shanghai, 200032 China

**Keywords:** Nasopharyngeal carcinoma, Tumor microenvironment, Immune cell, Immunotherapy

## Abstract

Nasopharyngeal carcinoma (NPC) arises from the epithelial cells located in the nasopharynx and has a distinct geographic distribution. Chronic Epstein-Barr virus (EBV) infection, as its most common causative agents, can be detected in 100% of NPC types. In-depth studies of the cellular and molecular events leading to immunosuppression in NPC have revealed new therapeutic targets and diverse combinations that promise to benefit patients with highly refractory, advanced and metastatic NPC. This paper reviews the mechanisms by which NPC cells to circumvent immune surveillance and approaches being attempted to restore immunity. We integrate existing insights into anti-NPC immunity and molecular signaling pathways as well as targeting therapies in anticipation of broader applicability and effectiveness in advanced metastatic NPC.

## Background

The stroma of NPC is densely infiltrated by immune cells and is therefore likely to elicit a specific adaptive antitumor response [1–4]. Conventional chemotherapy (IMRT singly or in combination with chemotherapy) is the standard treatment paradigm for early-stage NPC or locoregionally advanced NPC but is less effective in advanced recurrent or metastatic NPC (R/M NPC) due to nonspecific clinical signs and lack of early detection biomarkers [5]. Recently, immunotherapy in combination with chemotherapy is expected to be the first-line treatment option to extend progression-free survival of patients with R/M NPC [6, 7].

Recent single-cell sequencing delineates complex TME and crosstalk between cells. Persistent EBV infection decreases the immunogenicity of cancer cells and alters the tumor microenvironment [5, 8–10]. Although many factors that promote immune escape have been illuminated, treatment strategies to retrieve antitumor immunity have not been achieved. This review will systematically describe the ability of NPC to reprogram the TME through molecular and cellular interactions and shed light on the cutting-edge immunotherapies currently underway.

### Immune landscape of EBV-NPC

**Fig. 1:**
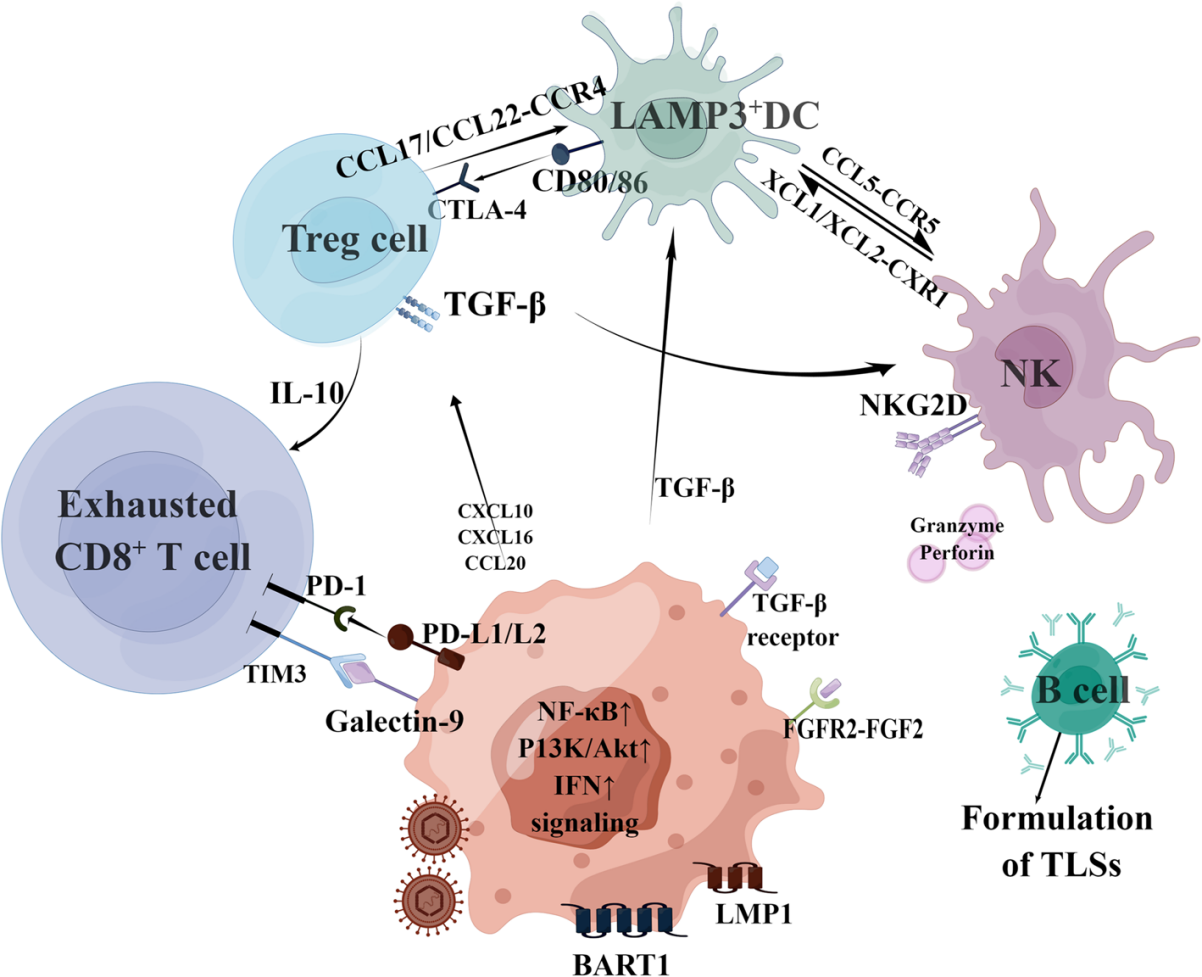
Mechanisms of immune escape in nasopharyngeal carcinoma. EBV-encoding miRNA mediates the escape of the NK cell killing by down-regulating the expression of ligands to NKG2D. NPC cells also secrete factors such as TGF-β to inhibits the recruitment of APCs, and meanwhile, secret several chemokines to recruit immunosuppressive regulatory T cells (Treg). Tregs further inhibit the function of APCs through the binding of CTLA-4 to CD86 and releases suppressive cytokines such as IL-10 to activated effector T cells (Teff), prohibiting their cytotoxicity to NPC. Its expression of membrane-bound TGF-β also inhibits the action of NKs. NPC cells directly inhibit the action of Teff by expressing PD-L1 ligand, which induce T cell anergy upon binding to PD-1. Secretion of exosomes containing LMP1, miRNA, Galectin1 and Galectin 9 leads to disfunction of Teff and NK

CD45 ^+^ immune cells existing in the NPC microenvironment mainly included T cells, B cells, natural killer cells (NK cells), and myeloid-derived suppressor cells (MDSCs) [11]. EBV in NPC cells exists in a type II latent state, encoding very limited non-coding RNAs and oncogenic proteins to reduce immunogenicity [12, 13], so lymphocytes fail to kill tumor cells despite the presence of massive infiltration in the tumor microenvironment (TME) (Fig. 1). Controlling persistent EBV infection and unlocking the potential of immunity requires induction of stable and effective T cell pools to prevent a recurrence. TCR analysis has confirmed the dynamic transition of T cells from activation to dysfunction [11], which is triggered early in tumor initiation, even before malignant transformation [14, 15]. IFN-induced genes are also upregulated by EBV infection, including ISG15, IFI6, IFI44L, and IFITM3. Long-term and sustained IFN activation may lead to T cell response failure [16, 17]——a mechanism that requires further investigations. EBV-infected tumor cells are usually resistant to NK cell surveillance. There have been many studies examining the role of NK cells on EBV-infected B cells, but for now, the studies on the interaction between NK cells and NPC cells is not well understood [18–20]. However, it is worth noting that tonsil NK cells may have an important effect on limiting EBV infection and subsequent carcinogenesis as the tonsils are the direct entry point for EBV [21].

Whole-exome sequencing studies have shown that misregulation of NF-κB signaling pathway is a major oncogenic driver of NPC. Besides, radiation-resistant nasopharyngeal carcinoma tissues often exhibit high levels of cancer-associated fibroblasts (CAFs), which produce substances that affect the biological behavior of nasopharyngeal carcinoma [22]. Fibroblast growth factor 2 (FGF2), secreted by CAFs, is the upstream molecule of the PI3K/AKT signal pathway. Aberrantly activated FGF signaling is involved in the accumulation of tumor-infiltrating MDSCs and M2-TAMs [23], remodeling the metabolism of NPC cells and promoting metastasis. Therefore, FGF2/FGFR2 may be a crucial target in NPC [24]. FGF/FGFR inhibitors promote further activation and infiltration of effector T cells into the TME by normalizing tumor vasculature. In this context, the combination of FGFR-targeted drugs with ICI may serve as an attractive therapeutic option to overcome to some extent the resistance to immunotherapy in NPC [25].

The increased B cells in NPC TME is conducive to the formulation of tertiary lymphoid structures (TLSs), in which B cells cooperate with T cells in maturation and activation [26, 27]. A high expression of B cell-related markers is associated with prolonged progression-free survival in NPC patients [11]. In addition, plasma cells are present within the TLSs, and deposits of many types of antibodies can be detected on tumor cells. TLSs have been found to produce an effective adaptive immune response like SLOs and are associated with longer survival in NPC patients [28]. Several approaches are being developed to induce TLSs formation using chemokines, cytokines, antibodies, antigen-presenting cells (APCs), or synthetic scaffolders [29].

Macrophages infiltrating the NPC microenvironment present an M1-M2 coupling paradigm and express both M1 and M2 markers [11]. Multicellular communication between macrophages and lymphocytes are prevalent but still unclear due to dynamic functional changes. Also, NPC cells can inhibit DCs maturation and IL-12 production, and promote the production of immunosuppressive factor IL-10 [30]. LAMP3 + DCs are highly mature DC cells that highly express suppressor proteins, produce multiple cytokines to recruit regulatory T cells (Tregs), and inhibit effector T cell functions [31]. The role of highly enriched MDSCs in cancer progression remains poorly understood. Functional analyses at both cellular level and animal level are necessary to clarify their modulatory potentials in NPC. Regulatory T cells (Tregs) will be recruited into the tumor microenvironment by substances secreted by the tumor and also assist the tumor to escape from immune surveillance. Tregs express immune checkpoint molecules in direct contact with other cells (e.g., LAMP3 + DCs), secret suppressive cytokines into the microenvironment, and also secret IDO to participate in metabolic reprogramming [32] (Fig. 1).

### NPC crosstalk leading to immune escape

#### Direct cell–cell contact

i. **Expression of inhibitory ligands/receptors**

Depleted T cells are characterized by overexpression of checkpoint molecules (e.g., PD-1, CTLA4, LAG3, TIM3, and TIGIT). Thus, depletion program might be elicited by EBV-mediated crosstalk with NPC cells [33–35]. EBER1 [36], miR-BART6-3p [37], circBART2.2 [38], and LMP1 [33] encoded by EBV activate the RIG-I pathway and upregulate PD-L1 expression on NPC, thus affecting T cell immune recognition and ultimately promoting immune escape. EBV-miR-BART11 and EBV-miR-BART17-3p promote the transcription of PD-L1 by targeting FOXP1 and PBRM1, respectively [39]. Immune checkpoint molecules expressed by NPC cells or immune cells constitute the rationale for the development of checkpoint inhibitor therapies, such as pembrolizumab, nivolumab, and camrelizumab, which are now used as standard second-line agents in the management of R/M NPC [7, 40]. Unfortunately, NPC cells can use alternative mechanisms, such as upregulating TIM-3 and LAG-3 to maintain immunosuppression [41, 42].

NKs can target EBV-infected cells that are in the lytic replication phase and maintain the homeostasis of the host immune system [43, 44]. A recent study found that LMP2A inhibited NK cell function through platelet aggregation by platelet factor 3, which offers a novel candidate for the development of NK cell immunotherapy [45]. EBV miRNAs have been confirmed to mediate the escape of NK cell killing by down-regulating NKG2D ligand and target pro-apoptotic proteins such as PUMA to resist NK cell cytotoxity [46–49]. Elevated pro-inflammatory cytokines IL-18 can also be mediated by EBV to induce PD-1 expression responsible for the functional exhaustion of NK cells [50].

ii. **HLA epitopes mutation**

Somatic MHC class I gene aberrations occur in greater than one-third of EBV-associated NPC [51], which may allow these NPC cells to evade the immune response [52]. Moreover, these alterations were significantly associated with prognostic indicators of patients. MHC class II gene alterations may also mediate the immune escape in NPC [53]. The mechanism by which MHC-I or MHC-II aberrations or both directly contribute to immune evasion remains to be determined. EBV-encoding miRNAs impair MHC class I/II- restricted antigen presentation [54] and reduce surface expression of HLA-B molecules, which are major MHC-I restriction elements of EBV-specific CD8 + T cell responses [55]. LMP2A also downregulates HLA-A/B/C and MIC-A/B expression through promoter hypermethylation, thereby inhibiting T and NK cell responses [56].

#### Indirect interactions between cells

i. **Exosomes**

**Fig. 2:**
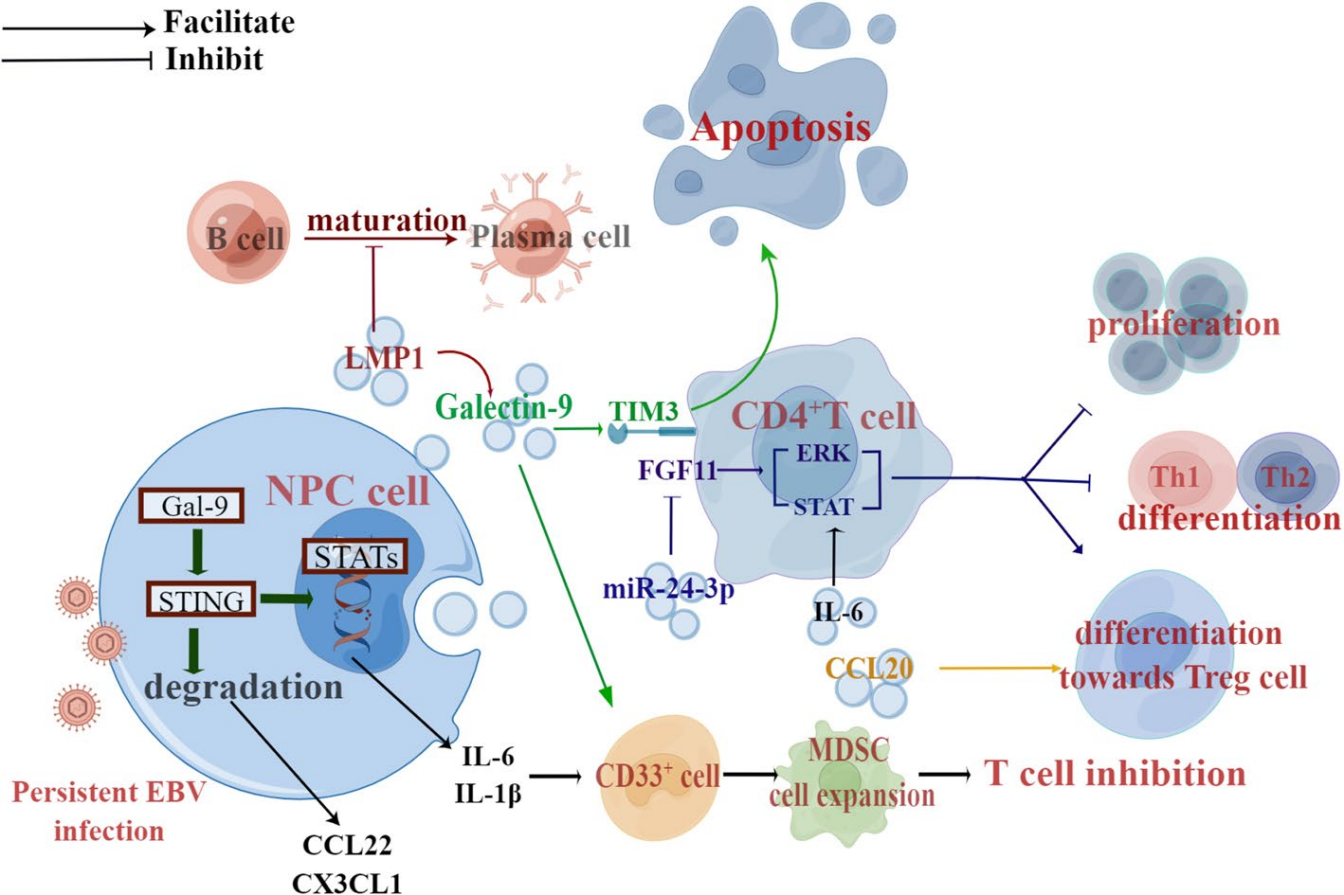
Pathways of exosomes involved in NPC immunosuppression. NPC affects the activities of immune effector cells by secreting exosomes to maintain persistent EBV infection and cause immunosuppression

Exosomes can play a key role as messengers to mediate cellular communication and deliver components to receptor cells. NPC cells indoctrinate exosomes (NPC-Exo) containing LMP1, and miRNAs to the TME, resulting in immune escape from host surveillance [32, 57] (Fig. 2). Mrizak et al. first identified the unique immunomodulatory ability of NPC-Exos to recruit Tregs into the microenvironment via the chemokine CCL20 and to recruit conventional CD4 + T cells and induce their conversion to inhibitory Tregs [32]. Exosomal LMP1 effectively activates oncogenic NF-kB and MAPK/Akt signaling [58, 59] and leads to T cell anergy [60]. They also severely inhibit the maturation of B lymphocytes into plasma cells [61]. In addition, exosomal LMP1 activates CAFs and then promotes tumor progression through stroma-tumor inter-relationships [62]. IL-6 is an inflammatory factor that promotes the occurrence of tumors. NPC-Exos show a more robust ability to induce IL-6 secretion from macrophages than exosomes derived from normal epithelial cells, and this difference is likely due to the different cargoes loaded [63].

The microenvironment characterized by hypoxia increases the level of exosomal miR-24-3p in NPC cells, thus enhancing the inhibitory effect on T cell proliferation and differentiation, and causing Treg-mediated suppression though inhibition of FGF11 [64]. In addition, MHC-I MICA and MICB ligands are likely to be removed by tumor cells into exosomes to evade recognition and clearance by NKG2D-activating receptor on natural killer cells and patrolling cytotoxic CD8 + T cells [65]. Understanding the specific substances that tumor cells select into exosomes can help reveal the mechanisms of immune evasion. As for NPC-lncRNAs, lncRNA-LINC00460 and lncRNA PVT1 have been involved in glucose metabolism and susceptibility to T cell-mediated lysis of NPC cells [66–68]. LncRNA TP73-AS1 is carried from NPC cells by extracellular vesicles and is associated with the polarization of macrophage toward the M2 phenotype [69]. TP73-AS1 is associated with a variety of cancers, so presumably, TP73-AS1 may be an NPC potential target [70–72].

Exosomal Galectin-9 produced by NPC cells stimulates the differentiation of MDSC by inhibiting STING signaling pathway and then upregulating a subset of cytokines, such as IL-1, IL-6, CX3CL1 and CCL22 [73–75]. Moreover, it can affect NK cell control of EBV and participate in M2 polarization of macrophages [76–78]. The binding of Galectin-9 to TIM-3 induces apoptosis in tumor cells, but for those PD-1-positive cells, PD-1 may inhibit this process. This is a novel mechanism of PD-1 that leads to exhausted T cell persistence [79]. High levels of soluble and exosomal Galectin-9 correlate with a higher risk of recurrence in NPC [80]. Unlocking more biological functions of the Galectin-9 will enable us to identify the its possibilities as a circulating marker and as a new target for immune checkpoint inhibitor therapy.

Recently, PD-L1 has been reported to be highly expressed in tumor-derived exosomes (Exo-PD-L1) that can directly contact T cells [81, 82]. Moreover, Exo-PD-L1 will suppress distant (e.g., spleen) T cells or even circulating T cells. It has been considered as a biomarker for HNSCC disease progression and clinical staging [83], as well as a predictor of immunotherapy [84]. It’s reasonable to assume that in addition to EBV DNA, PD-L1 in exosomes also has predictive value as a liquid biopsy method. Notably, Exo-PD-L1 can be secreted in large amounts by irradiated tumor cells, which suggests that the immunomodulatory effects of tumor-derived exosomes in response to radiotherapy and a synergistic therapeutic effect of radiotherapy and immune checkpoint inhibitors for R/M NPC [85]. Future work should identify more co-expressing molecules on PD-L1-positve exosomes to better predict the immunotherapy for NPC and other HNSCCs.

ii. **Cytokines and chemokines**

Cytokines such as IL-6, IL-8, IP-10, TNF-α, VEGF, EGFR, and MIP-3α were found to be elevated in nasopharyngeal carcinoma [86–88]. NPC cells induce macrophage polarization toward an M2-like phenotype by secreting ISG15 [42]. In addition, EBV-infected NPCs undermine the immune response by the simulation of regulatory T cells that secrete IL-10, which inhibits T cell and NK cell infiltration and IFN-γ secretion [89]. LMP1 regulates the production of such chemokines as CXCL9, CXCL10, CX3CL1, and CCL20 through constitutive activation of the NF-κB pathway, leading to T lymphocyte infiltration, but they also inhibit cytotoxic T cells through PD-L2/PD-1 interactions [87, 90]. LMP1 also contributes to MDSC expansion through metabolic reprogramming leading to the release of IL-1β, IL-6, and GM-CSF [91]. IL-6 and TNF-α can be secreted by viral oncoproteins produced by NPC. It was found that baseline levels of IL-6 and TNF-α were adversely correlated with 2-year survival in NPC patients, which may be mediated by the immunosuppressive effects of IDO [92].

iii. **Metabolic mediators**

**Fig. 3:**
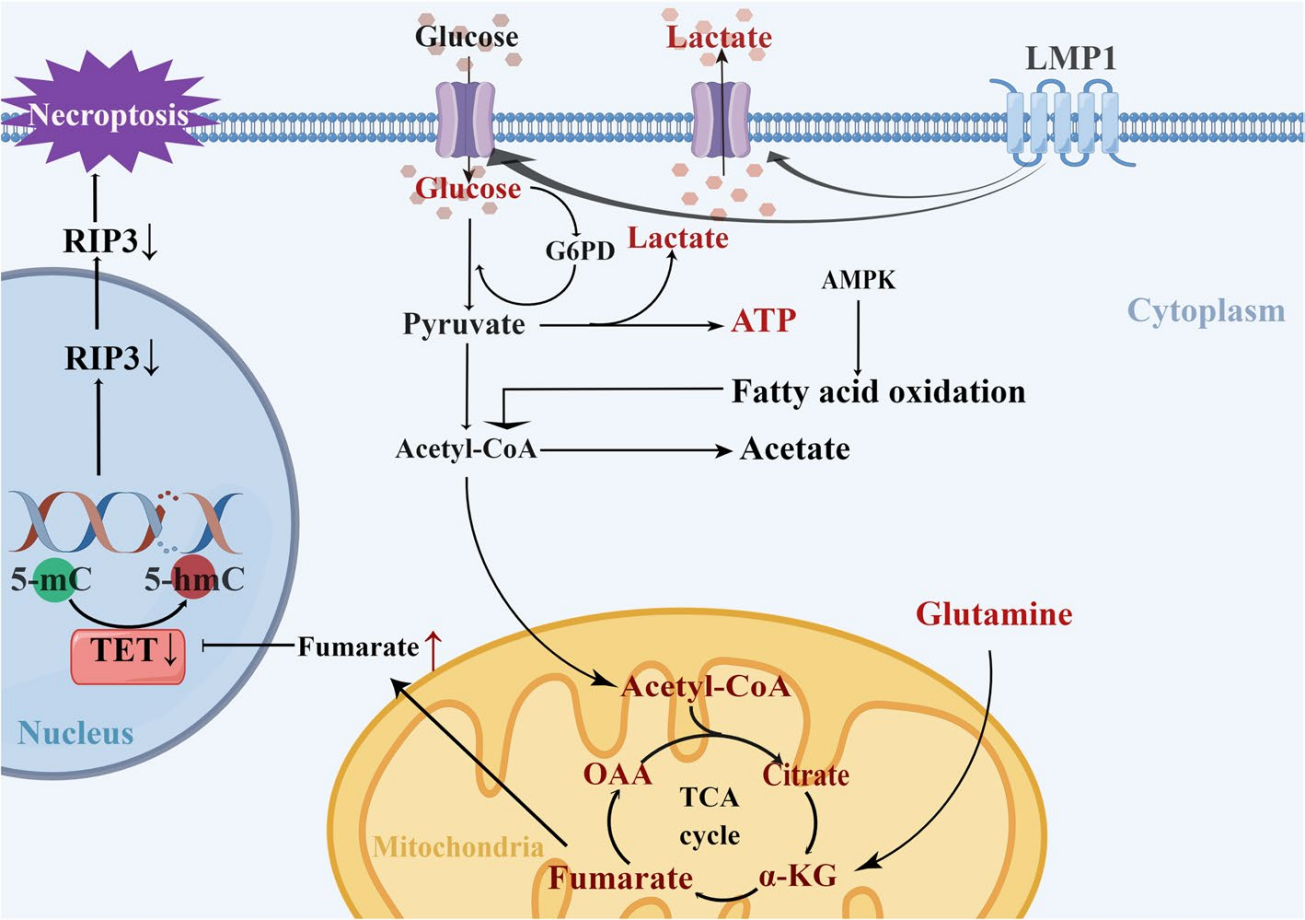
Aerobic glycolysis in NPC cells. LMP1 secreted by EBV enhances glucose consumption and lactate generation in NPC cells. Increased glucose consumption in the tumor cells leads to immune cell starvation. EBV (LMP1) leads to the accumulation of fumarate in the tricarboxylic acid cycle (TCA), which inhibits the expression of RIP3 and protects NPC cells from TNF-induced necrotizing apoptosis. GLUT1: glucose transporter 1, OAA: Oxaloacetic acid, a-KG: a–ketoglutarate, LDH: lactate dehydrogenase, G6PD: glucose-6-phosphate dehydrogenase

There are a growing number of studies on metabolic reprogramming and immune evasion of NPC cells, but the majority have focused only on one side of the story. Regarding the competition for nutrients, NPC cells have a greater competitive advantage over immune cells for glucose, thus inhibiting the activity of immune cells [93, 94] (Fig. 3). LMP1 activates multiple cellular signaling pathways in NPC cells [95], among which the FGF/FGFR signaling pathway is activated to increase glucose and glutamine uptake, LDHA activity, lactate generation, and the level of hypoxia-inducible factor 1 (HIF-1α) [96]. Interestingly, glutamine deprivation enhanced glucose uptake by tumor cells, thereby reducing the amount of glucose available to immune cells, suggesting that glutamine may be the limiting factor in TME, rather than glucose. Selective cellular partitioning of nutrients could be used to develop novel therapies that target specific cell types as well as more accurate imaging strategies [97].

Given the high metabolic rate of cancer cells, lactic acid secreted by tumors is an important metabolite in TME [98]. High concentrations of lactate interfere with the metabolism of immune effector cells, promote conversion to an immunosuppressive phenotype, inhibit the release of pro-inflammatory cytokines, and activate CAFs to produce VEGF to stimulate angiogenesis [99–102]. Similarly, lipid metabolism also affects T cell function and the expression of inhibitory receptors [103]. Fatty acids (FAs) have been reported to facilitate the malignant behavior of tumor cells [104, 105]. Virus-encoded proteins reprogram cellular metabolism and promote the optimal generation of progeny virion generation [106]. The LMP1 leads to the accumulation of the metabolite fumaric acid, inhibiting the expression of RIP3 and protecting NPC cells from TNF-induced necrosis secreted by macrophages [107]. Targeting lipid metabolism may be a novel idea worthy of further study in the treatment of NPC.

Other oncological metabolites such as adenosine, kynurenine, and tryptophan can affect tumor immunity. Elevated HIF enhances the expression of external nucleosidase (CD39 and CD37), which convert ATP to adenosine, a metabolite of immunosuppressant mediators. Extracellular adenosine attenuates T cell adhesion and cytotoxicity through A2a and A3 adenosine receptors [108, 109]. IDO is highly expressed in NPC, and as previously mentioned, IDO is an immunosuppressive metabolite that catabolizes intracellular tryptophan to produce kynurenine. The increased kynurenine in the microenvironment acts on the AHR signaling axis of the naïve T cells, causing them to differentiate into Tregs [110]. In addition, IDO can directly activate Treg cells via the PD-1/PD-L1 binding. There are clinical trials actively investigating IDO inhibitors in combination with immunotherapy (NCT03823131) or other chemotherapies [111]. Other metabolic inhibitors such as 3-bromopyruvate and oxalate inhibit hexokinase (HK) and LHDA activity and activate macrophage and T cell in animal models including NPC [112, 113]. Therefore, the combination of small molecule drugs or antibody intervention drugs that target metabolic processes with immunotherapy agents may become a potential clinical choice.

### Augmenting or rescuing immunity in NPC

The combination of radiotherapy and chemotherapy (gemcitabine, cisplatin, and fluorouracil) is the conventional treatment paradigm for NPC but face obstacles in treating locoregional advanced or metastatic NPC. It is urgent to seek more effective strategies with greater efficacy and less toxicity [114, 115]. Several approaches including checkpoint inhibitors and cell-based immunotherapy have been used to revitalize the depleted immune cells in the NPC TME (Table 1).

**Table 1:** Immunotherapies for NPC treatment

Target	Effects	Treatment	NCT number
PD-1	Bypass immune checkpoint	Nivolumab, Pembrolizumab, Toripalimab, Penpulimab, Camrelizumab, Sintilimab, Tislelizumab, Treprilimab, SHR-1701	NCT02054806 [7], NCT02339558 [40], NCT02915432 [116], NCT03581786 [6], NCT03558191, NCT03267498, NCT03544099, NCT03734809, NCT03707509, NCT03925090, NCT03924986, NCT04282070, NCT04376866, NCT04447612, NCT04421469, NCT04534855, NCT04736810, NCT04833257, NCT04917770, NCT04944914, NCT04978012, NCT3707509, NCT05020925, NCT05097209, NCT05341193
PD-L1	Bypass immune checkpoint	SHR-1701, Avelumab	NCT04282070, NCT05020925, NCT04562441
CTLA-4 & PD-1	Bypass immune checkpoint	Ipilimumab, AK104, IBI310	NCT03097939, NCT04220307, NCT04945421
LMP2	Direct cytotoxicity	LMP2 Antigen-specific TCR T cell	NCT03925896
EBV	Direct cytotoxicity	Autologous EBV specific Cytotoxic T cell	NCT03648697, NCT02287311, NCT02578641 [117]
TGF-β	Direct cytotoxicity	TGF-β Resistant CTLs	NCT02065362

*Abbreviations*: *CTLA-4* Cytotoxic T-lymphocyte antigen 4, *PD-1* Programmed death 1, *PD-L1* Programmed death ligand 1, *CAR* Chimeric antigen receptor

#### Checkpoint inhibitors

Checkpoint inhibitors act on chronically stimulated T cells to reverse the fixed “exhaustion” state. About one-third of NPC cells and half of immune cells express PD-L1 [118, 119], and the difference in expression ratios among patients correlated strongly with the clinical effect of anti-PD-1/PD-L1 immunotherapy. NCI-9742 is the first completed report on the clinical response of Nivolumab in R/M NPC patients with an ORR of 20.5% and a 1-year OS rate superior to historic data in similar populations [40]. Like Nivolumab, pembrolizumab demonstrated a favorable antitumor activity in R/M NPC patients [7]. However, more impressive is the median PFS of 11.7 months with the addition of Toripalimab to GP chemotherapy as a first-line treatment for R/M NPC patients [6]. RATIONAL-309 studies (NCT03924986, Phase III) also showed that by April 2022, Tislelizumab plus GP significantly extended the median PFS of R/M NPC patients compared to placebo plus GP (NR vs 13.9 months) after a follow-up of 15.5 months. Currently, there are lots of clinical trials testing checkpoint inhibitors alone or in combination with chemotherapy, radiotherapy and other immunotherapies (Table 1).

Another predominant regulatory checkpoint is TIM3. Inhibition of its ligand, Galectin-9, selectively revigorated infiltrating T lymphocytes by interfering with the interaction between PD-1 and TIM3 [79]. As Galectin-9 is specifically expressed by NPC cells, the TIM3/Galectin-9 interaction represents a promising approach to overcoming the resistance to the PD-1/PD-L1 inhibitors. The checkpoint receptor found on the surface of Tregs, CTLA4, binds CD80/86 on DCs to inhibit antigen presentation and secret IDO1 to induce the Treg proliferation [31]. The efficacy of the combination of ipilimumab/nivolumab in NPC are currently under investigation in phase II clinical trial (NCT03097939). Preliminary data up to November 2020 showed a partial response rate of 35% with a median response duration of 5.9 months, synergistically enhancing the efficacy of PD-1 monotherapy. Other therapeutic targets, such as LAG3, TIM3, and TIGIT, are currently immature compared to PD-1, but it is reasonable to assume that combining these molecules would be a more effective treatment option for patients with R/M NPC.

#### Expanding EBV/NPC-specific immune cells

An ongoing Phase III study (NCT02578641) using chemotherapy (Gemcitable & Carboplatin) followed by autologous, vitro-expanded EBV-specific cytotoxic T cell (EBVST) as first-line treatments for R/M NPC patients achieves a promising result with an overall response rate of 71.4% [117]. The individuals who received EBVST immunotherapy but showed no benefit were due to the expansion of mMDSCs leading to a dominant immune-suppressive effect, which points to a window of therapeutic opportunity [120]. Similar studies are being conducted using adoptive cellular-based immunotherapies such as EBV-TCR-T (NCT03648697) and LMP, BARF1 & EBNA1 Specific CTL (NCT02287311).

Other emerging cellular immunotherapy strategies, such as chimeric antigen receptor NK-Cell Immunotherapy (CAR-NK) and CAR-Macrophages (CAR-M), should be evaluated in NPC for their promising clinical efficacy in other cancers [121, 122]. Cancer vaccine, on the other hand, represents active immunotherapy that delivers tumor-specific antigens through APCs or viral vectors to improve immune system recognition. DCs expressing LMP2 and recombinant modified vaccinia Ankara vaccine (MVA) have been shown to be safe and well-tolerated [123]. Customized therapeutic vaccines can also be augmented by checkpoint inhibitor therapies [124]. Hence, future studies could consider combination immunotherapy to enhance the clinical responses, for example, immune checkpoint blockade combined with adoptive T-cell therapy [125, 126] or combining CAR T cells therapy combined with Ankara-oncolytic virus [127].

#### Targeting exosomes

There is substantial clinical evidence that exosomes can be used as biomarkers of disease, and a number of drugs have been developed to reduce the secretion of exosomes released by cancer cell and their effect on immune cells, such as RAB27A inhibitors, nSMase inhibitors, PPIs and calcium channel blockers [128]. Some of these inhibitors only affect exosomes released by tumor cells, which provides ideas for inhibiting the release of NPC-Exos. One of the most significant characteristics of NPC-Exos is the high level of miRNA content that governs the reprogramming of immune active factors and immune cell functions [129]. The use of exosome inhibitors or corresponding miRNA inhibitors might be able to block their effect of NPC cells on immune cells [130].

Besides focusing on NPC-Exos, exosomes secreted by immune cells can also serve as powerful anti-cancer weapons, such as NK-derived exosomes and Dendric cell exosomes [131, 132]. In particular, exosomes derived from phosphor-antigen-expanded Vδ2-T cells can kill EBV-infected cells more efficiently [133]. In recent years, vaccines targeting exosomes have also been exploited, for example, dual-acting exosome vaccines for melanoma and lung cancer have showen the effectiveness in mouse models. However, exosomal vaccines have not been specifically tested in NPC [134]. But there are already many EBV vaccines that can be genetically engineered by exosomes to express specific antigens or target tumor cells to increase immunogenicity [135].

#### Targeting cytokine and chemokine

Immunomodulatory compounds such as cytokines and chemokines are being employed to reshape the tumor immune microenvironment. EBV-NPC cells produce high levels of TGF-β to promote a more aggressive malignant phenotype. TGF-β signaling is a crucial mediator not only of suppressive phenotypic transformation of tumor-infiltrating lymphocytes but also of remodulation in the stromal environment [136–138]. Given the axial role of the TGF-β signaling pathway in tumorigenesis, it can be regarded as an attractive target for EBV-NPC therapy. Currently, a clinical trial targeting TGF-β resistant T cells is being explored (NCT02065362).

Many NPC patients are accompanied by high EGFR expression, which often predicts an aggressive phenotype. LMP-1 can induce the expression and activation of EGFR and its ligand TGF-α in epithelial cells. Numerous studies have indicated that EGFR signaling plays a vital role in NPC pathogenesis [139–145]. LMP1 can also activate the insulin-like growth factor 1 receptor (IGF1R), which promotes epithelial cell carcinogenesis and cause excessive anti-autophagic signaling to suppress anticancer immunosurveillance. It may constitute a druggable target for NPC in combination with chemo-immunotherapies [146, 147]. Altogether, further investigations on molecular targets are warranted for the treatment and management of NPC.

## Conclusions

A deeper understanding of the crosstalk between the immune system and NPC that leads to suppression of anti-tumor response will greatly aid in developing more effective therapies. The identification of cytokines, immunosuppressive cell subpopulations and checkpoint pathways has allowed many immunotherapeutic drugs to be tested in clinical trials and hopefully used in clinical practice. The heterogeneity and complexity of advanced NPCs undoubtedly require a combination of these drugs to establish durable, lifelong immunity. In addition, consideration must be given to the optimal dose needed to establish durable tumor control while minimizing adverse events. Also, the unique characteristics of each NPC patient must be determined before treatment to select a balanced combination that maximizes PFS and minimizes toxicity.

## Data Availability

Not applicable.

## Abbreviations

R/M NPC: Recurrent or metastatic NPC
EBV: Epstein-Barr virus
NK cells: Natural killer cells
MDSCs: Myeloid-derived suppressor cells
TME: Tumor microenvironment
ISG15: Interferon stimulating gene 15
IFI6: Interferon-inducible-protein 6
IFI44L: Interferon-inducible-protein 44
IFITM3: Interferon—induced transmembrane protein 3
FGF: Fibroblast growth factor
TLSs: Tertiary lymphoid structures
APCs: Antigen-presenting cells
CAFs: Cancer-associated fibroblasts
PD-1/PD-L1: Programmed death-1/programmed death ligand-1
CTLA4: Cytotoxic T-lymphocyte antigen 4
TIM3: T cell immunoglobulin domain and mucin domain-3
LAG3: Lymphocytes activating gene 3
TIGIT: T cell immunoreceptor with Ig and ITIM domains
miRNAs: MicroRNAs
lncRNA: Long non-coding RNA
HLA: Human leucocyte antigen
MHC: Major histocompatibility complex
MIC: MHC class I molecular associated protein A/B
HIF-1: Hypoxia-inducible factor-1
EBNA: Epstein-Barr virus nuclear antigen
LMP: Latent membrane protein
FAs: Fatty acids
RIP3: Receptor interacting protein 3
IP-10: Interferon-inducible protein-10
MIP-3α: Macrophage inflammatory protein-3α
GM-CSF: Granulocyte–macrophage colony-stimulating factor
